# Expression and Functional Study of Extracellular BMP Antagonists during the Morphogenesis of the Digits and Their Associated Connective Tissues

**DOI:** 10.1371/journal.pone.0060423

**Published:** 2013-04-03

**Authors:** Carlos I. Lorda-Diez, Juan A. Montero, Joaquin Rodriguez-Leon, Juan A. Garcia-Porrero, Juan M. Hurle

**Affiliations:** 1 Departamento de Anatomía y Biología Celular and IFIMAV, Universidad de Cantabria, Santander, Spain; 2 Departamento de Anatomia, Universidad de Extremadura, Badajoz, Spain; Instituto Gulbenkian de Ciência, Portugal

## Abstract

The purpose of this study is to gain insight into the role of BMP signaling in the diversification of the embryonic limb mesodermal progenitors destined to form cartilage, joints, and tendons. Given the importance of extracellular BMP modulators in *in vivo* systems, we performed a systematic search of those expressed in the developing autopod during the formation of the digits. Here, we monitored the expression of extracellular BMP modulators including: *Noggin*, *Chordin*, *Chordin-like 1*, *Chordin-like 2*, *Twisted gastrulation*, *Dan*, BMPER, *Sost*, *Sostdc1*, *Follistatin*, *Follistatin-like 1*, *Follistatin-like 5* and *Tolloid*. These factors show differential expression domains in cartilage, joints and tendons. Furthermore, they are induced in specific temporal patterns during the formation of an ectopic extra digit, preceding the appearance of changes that are identifiable by conventional histology. The analysis of gene regulation, cell proliferation and cell death that are induced by these factors in high density cultures of digit progenitors provides evidence of functional specialization in the control of mesodermal differentiation but not in cell proliferation or apoptosis. We further show that the expression of these factors is differentially controlled by the distinct signaling pathways acting in the developing limb at the stages covered by this study. In addition, our results provide evidence suggesting that TWISTED GASTRULATION cooperates with CHORDINS, BMPER, and NOGGIN in the establishment of tendons or cartilage in a fashion that is dependent on the presence or absence of TOLLOID.

## Introduction

In living organisms chondrogenesis occurs in the context of complex morphogenetic processes associated with the formation of other connective tissues [1]. The formation of digits in the developing vertebrate limb illustrates this phenomenon. In the embryonic limb autopod, mesodermal cells that share a unique origin from the lateral mesoderm, form phalangeal cartilages and the associated perichondrium, interphalangeal joints and tendons. Hence, in terms of tissue differentiation, the formation of a digit, includes the following: formation of and subsequent differentiation of prechondrogenic condensations; differentiation of the perichondrium; the formation and subsequent differentiation of tendon blastemas, including the establishment of the entheses (i.e., the zone where the tendon attaches to the bone primordia); and the formation of joints (hyaline articular cartilage, synovium, and fibrous capsule). Different BMP genes exhibit regulated expression patterns in the undifferentiated and interdigital mesoderm, joints and tendon blastemas [2], [3]. There is compelling evidence that BMP signaling plays a key role in each of the aforementioned morphogenetic events [4]–[11]. However, the mechanism by which BMPs function to establish divergent cell fates during developmental processes such as chondrogenesis, joint differentiation or tenogenesis while using the same population of cell progenitors remains to be clarified [5]. In avian limbs, the overexpression of BMPs results in a dramatic increase in chondrogenesis [12]–[14], and loss-of-function experiments cause severe skeletal truncations [14]. In mammals, the alterations observed in naturally occurring or experimentally induced genetic mutations of members of the BMP signaling pathway, confirmed its role in skeletogenesis [4], [5], [7], [15]–[20]. However, digit phenotypes are not very informative, which is most likely due to the functional redundancy of these molecules [16], [21].

In the canonical signaling pathway, active BMPs are released in the extracellular space and subsequently bind to transmembrane type I and type II serine-threonine kinase receptors, triggering an intracellular cascade that results in the phosphorylation and nuclear translocation of Smad 1/5/8 proteins, which in conjunction with Smad 4 and other transcription factors, regulate target gene expression. In addition, BMPs activate non-Smad pathways that involve signaling via mitogen-activated protein kinases (MAPK; see [22]). Taking into account that the signaling cascade activated by the different BMPs is constant, it is believed that the differences in the response of BMP target cells may reside largely on the intensity of the signal. Hence, gradients of BMP signaling play a key role during vertebrate development to establish the dorso-ventral axis of the gastrula, and most likely, function in other embryonic models [23], [24]. The morphogenetic gradient relies largely on the functional interactions between BMPs and secreted BMP-binding molecules, often called “extracellular BMP antagonists” (see: [25], [26]).

Several BMP antagonists are expressed at advanced stages of limb skeletogenesis [27]–[29]. In the course of digit development, Noggin [14], Chordin [30], Chordin-like 1 [31], [32], BMPER [33], Sost [34], Sostdc1 [35], [36], Dan [37], [38], Follistatin [39], [40], and Follistatin-like 1 [41] have been detected; however, except for Noggin [4] and Follistatin-like 1 [42], mice that are mutant for these factors lack a digit phenotype. This lack of a phenotype is indicative of intense functional redundancy. Therefore, an appropriate understanding of the role of BMP antagonists in digit morphogenesis requires a comprehensive analysis of the BMP modulators that are expressed in the course of digit formation. The goal of this study was to analyze the involvement of extracellular modulators of BMP signaling during the early differentiation of the structural components of the embryonic digits. In an initial systematic gene expression study we identified 13 different BMP antagonists with dynamic expression patterns associated with the differentiation of the phalanges, interphalangeal joints and tendons. The role of these factors and their regulation by major signaling pathways that are involved in limb morphogenesis was next explored through gain-off-function experiments in high-density cultures of digit mesodermal progenitors. Our findings provide new insights that clarify the role of BMPs in the divergent differentiation of the connective tissue progenitors into cartilage, tendon, and joint tissues, which is a process of major importance in regenerative medicine of the locomotor apparatus.

## Materials and Methods

In this work, we employed Rhode Island chicken embryos from day 4,5 to day 8 of incubation (id) equivalent to stages 24 to 32 HH. This study was approved by the Cantabria University Institutional Laboratory Animal Care and Use Committee and carried out in accordance with the Declaration of Helsinki and the European Communities Council Directive (86/609/EEC).

### In situ Hybridization

In situ hybridization was performed in 100 µm vibratome sectioned specimens. Samples (a minimum of 5 sectioned limbs of each stage) were treated with 10 µg/ml of proteinase K for 20 minutes at 20°C. Hybridization with digoxigenin labeled antisense RNA probes was performed at 68°C. Alkaline phosphatase-conjugated anti-digoxigenin antibody (dilution 1∶2000) was used (Roche). Reactions were developed with BM Purple AP Substrate precipitating (Roche). No variability among the samples was appreciated in the expression pattern of the analyzed genes was constant in all the samples analyzed.

The probes for *Noggin*, *Chordin (Chd*), *Chordin-like 1* (*Chdl-1*; *ventroptin*), *Chordin-like 2* (*Chdl-2)*; *Twisted gastrulation* (*Tsg*; *Twsg 1*); *DAN* (*differential screening-selected gene aberrative in neuroblastoma*); *BMPER* (*BMP binding endothelial regulator; crossveinless 2*), *Sost* (*Sclerostin*), *Sostdc1* (*Sclerostin domain containing-1; Uterine sensitization associated gene-1; Wise; Ectoidin*), *Follistatin (Fst)*, *Follistatin-like 1* (*Fstl-*1; Flick), *Follistatin-like 5 (Fstl-5)*, *Tolloid* (Tll1; *Colloid*; *Tolloid-like 1*) and *Alk1* were obtained by PCR using the following primers: for *Noggin,* 5′- aaggatggatcattcccagt-3′ and 5′-ctagcaggagcacttgcact-3′; for *Chd,* 5′-acctgctcttctccatcagc-3′ and 5′- ccatagtgatgttggcatgg -3′; for *Chdl-1,* 5′-ggaattccgatgagaagaaagtggagatcg-3′, and 5′-gctctagagcagattcaccgtgggagtat -3′; for *Chdl-2,* 5′- ggcaccactgtgaagatcg -3′ and 5′- tgtagttctgcgcttcttgc -3′; for *Tsg*, 5′- gtcagcaagtgcctcatcc -3′ y 5′- cttgcactgatgtattgacatgc -3′; for *Dan*, 5′- tgcgagtccaagtccatcc -3′ and 5′- ggctcttctacctcctgttgg -3′; for *BMPER*, 5′- aagcgagatgacctgattgg -3′ and 5′- cgctgaggacataggactgg -3′; for *Sost*, 5′- ctctgtctgcgtcctcatcc -3′ and 5′- taccgagtgtagcgcttgc -3′; for *Sostdc1*, 5′- ctccgccattcacttctacg -3′ and 5′- tgtgctgcctggtgtatcg -3′; for *Fst,* 5′- ccgtgtgtggcttagatgg -3′ and 5′- gagttgcaagatccagagtgc -3′; for *Fstl-1,* 5′- gaatgtgcagtgactgagaagg -3′ and 5′- tgagcagcttgttggtctcc -3′; for *Fstl-5,* 5′- tcagccactcataagattacgc -3′ and 5′- tcattggtgtccacaagtcc -3′; for *Tll1*, 5′- gaagatggagcctggagagg -3′ and 5′- acggaactcaatccacatcc -3′; and for *Alk1*, 5′- agcgactacctggacattgg -3′ and 5′-ccttcttcatgtcctcgaagc -3′. Chicken probes for *Bmpr1a and Bmpr1b* were kindly provided by Lee Niswander and for *Alk2* by Joan Massague.

The above mentioned BMP antagonists have been identified in the genome of most the analyzed vertebrates (*Homo sapiens, Pan troglodytes, Macaca mulatta, Mus musculus, Rattus novergicus, Gallus gallus, Xenopus laevis*) except for the zebrafish (*Danio rerio*) where there is only a *Chordin-like* [43], which is thought to represent *Chordin-like 1* and *Chordin-like 2* of mammals.

### Phospho Smad 1/5/8 and p-c-Jun Immunolabeling

Limb buds between 6 and 8 days of incubation were fixed in 4% PFA O/N at 4°C, washed in PBS and sectioned with a vibratome. Sections were incubated O/N at 4°C with the primary antibody. Specimens were next washed in PBS, incubated O/N in the secondary antibody washed for 2 h in TBS, dehydrated, cleared and examined with the confocal microscope (LEICA LSM 510). Polyclonal antibodies against phospho-SMAD1/SMAD5/SMAD8 (Ser463/465; Cell Signaling) and p-c-Jun (Sc-822, Santa Cruz Biotechnology) were employed. For double labeling purpose, we employed actin staining using 1% Phalloidin-TRITC (Sigma).

### Experimental Induction of Ectopic Digits

In vivo analysis of gene regulation preceding the formation of an ectopic digit was performed in samples of interdigital tissue 10, 14, and 20 hr after implantation at 5.5 id of heparin beads (Sigma) incubated for 1 hr in 2 µgr/ml TGFβ1 (R&D Systems). This treatment leads to the formation of ectopic digits detectable by alcian blue staining 20 hr or later after bead implantation [44]. The contralateral left limb or limbs treated with beads incubated in PBS, were employed as controls.

### Micromass Mesodermal Cultures

Progenitor mesodermal cells of the digit tissues were obtained from the progress zone region located under the apical ectodermal ridge of chick leg buds of embryos at 4.5 id (25 HH). Cells were dissociated and suspended in medium DMEM (Dulbecco’s modified Eagle’s medium) with 10% fetal bovine serum, 100 units/ml penicillin and 100 µg/ml streptomycin. Cultures were made by pipetting 10-µl drops of cell suspension at a density of 2.0×10^7^ cells/ml into each well of a 24-well plate. The cells were left to attach for 2 hr and then 200 µl serum-free medium was added. In gene overexpression experiments (see below) cultures were performed with DMEM medium containing 10% fetal bovine serum and 50 µgr/ml of ascorbic acid. We employed these cultures for analyzing the effects of adding BMP modulators on gene regulation, cell proliferation and cell death and to study the regulation of BMP modulators by major signaling pathways acting in the autopod.

The effect of BMP modulators and BMP2 were analyzed by adding recombinant protein to the medium in 24 hr cultures. Treatments were maintained for another 24 hr period. After testing different protein concentrations we selected the following: human recombinant BMP2 200 ngr/ml (Peprotech); human recombinant NOGGIN, 200 ngr/ml (R&D Systems); human recombinant CHDL-1, 2400 ngr/ml (R&D Systems); mouse recombinant CHDL-2 1200 ngr/ml (R & D Systems); human recombinant TSG 1000 ngr/ml (R & D Systems); mouse recombinant DAN 3000 ngr/ml (R & D Systems); Follistatin 800 ngr/ml (Peprotech). After these treatments we analyzed by Q-PCR changes in the expression of cartilage markers (*Sox9*, *type 2 Collagen*; and *Bmpr1b*), fibrogenic markers (*Scleraxis*, *type 1 Collagen* and *Tgfβ2*), and joint markers (*Activinβα*, *Gdf5*, and *Jaws*). The selected genes are well known markers of the corresponding morpho-developmental processes. Only *Jaws* has not been used very often as joint marker, but it has been shown that it is essential for the formation of interphalangeal joints [45].

To study the effect of autopodial signaling pathways in the expression of BMP modulators we performed 6 hr treatments to 48 hr Micromass cultures. We employed: FGF2 66 ngr/ml (Peprotech), ACTIVIN A 200 ngr/ml (Peprotech), all-trans-retinoic acid (RA) 50 ngr/ml (Sigma); BMP2, 200 ngr/ml (Peprotech); TGFbeta2, 10 ngr/ml (R&D Systems); and WNT5a 100 ngr/ml (R & D Systems).

### Cell Transfections

Gain-of-function experiments for *Tsg*, and *BMPER* were performed by overexpression constructs containing the mouse coding sequences. We employed “Addgene plasmid 25778” for *Tsg* and “Addgene plasmid 25776” for *BMPER* (both made by Dr Edward De Robertis). For *Fstl-1* overexpression we used a construct based on the coding sequence of the human gene cloned into the pCMV6-XL5 vector (Origene, MD, USA). For *Dan* overexpression we used a construct based on the coding sequence of the mouse gene cloned into the pCMV6-ENTRY vector (Origene, MD, USA) Control samples were transfected with empty plasmids. Limb mesodermal cells were electroporated employing the Multiporator System (Eppendorf) and cultured in high-density conditions as indicated above. After 48 hr of cultured the level of gene overexpression and the expression of cartilage, joint, and tendon markers were evaluated by Q-PCR.

### Flow Cytometry

Cell proliferation and cell death was deduced from measurement of DNA content by flow cytometry in control Micromasses and in Micromasses treated with CHDL-1, TSG, or both CHDL-1 and TSG. For this purpose cultures were dissociated to single-cell level by treatment with Trypsin EDTA (Lonza). 1 million cells (5 Micromasses) were used in each test. For propidium iodide (PI) staining the cells were washed twice in PBS and centrifuged at 405 g, 5 min at 4°C. The samples were then incubated overnight at 4°C with 0.1% sodium citrate, 0.01% TritonX-100 and 0.1 mg/ml PI. Cell suspension was subjected to flow cytometry analysis in a Becton Dickinson FacsCanto cytometer and analyzed with Cell Quest software. This technique allows the titration of apoptotic (hipodiploid) and proliferating (hiperdiploid) cells according to their DNA content deduced from PI staining [46].

### Real time Quantitative PCR (Q-PCR) for Gene Expression Analysis

In each experiment total RNA was extracted and cleaned from specimens using the RNeasy Mini Kit (Qiagen). RNA samples were quantified using a spectrophotometer (Nanodrop Technologies ND-1000). First-strand cDNA was synthesized by RT-PCR using random hexamers and M-MulV reverse transcriptase (Fermentas). The cDNA concentration was measured in a spectrophotometer (Nanodrop Technologies ND-1000) and adjusted to 0.5 µg/µl. Q-PCR was performed using the Mx3005P system (Agilent) with automation attachment. In this work, we have used SYBRGreen (Agilent) based Q-PCR. *Gapdh* had no significant variation in expression across the sample set and therefore was chosen as the normalizer in our experiments. Mean values for fold changes were calculated for each gene. Each value in this work represents the mean ± SEM of at least three independent samples obtained under the same conditions. Samples consisted of 4 Micromass cultures or 15 interdigital spaces. Data were analyzed using one-way analysis of variance followed by Bonferroni tests for post-hoc comparisons or Student’s *t* test, for gene expression levels in overexpression experiments. Statistical significance was set at p<0.05. All the analyses were done using SPSS for Windows version 18.0. Primers for Q-PCR are included as Supplementary Table 1.

**Table 1:** Regulation of BMP modulators.

	10 h	14 h	20 h
**Noggin**	1,00±0,1	1,93±0,2**	3,98±0,9*
**Chd**	1,36±0,3	1,11±0,1	1,23±0,4
**Chdl-1**	3,64±1,0*	3,83±0,7*	8,09±1,7*
**Chdl-2**	1,90±0,2*	2,33±0,4*	2,94±1,0**
**Tsg**	1,27±0,2	1,27±0,4	1,24±0,1
**Dan**	1,01±0,0	1,16±0,0	3,40±0,9*
**Bmper**	1,30±0,1	1,85±0,3*	1,85±0,2*
**Sost**	1,02±0,2	0,94±0,0	1,00±0,0
**Sostdc1**	0,92±0,1	1,13±0,1	1,12±0,0
**Follistatin**	9,09±1,3***	5,14±0,4***	8,16±1,7*
**Fstl-1**	0,86±0,0	1,15±0,0	1,12±0,0
**Fstl-5**	0,47±0,1*	1,38±0,2	0,99±0,1
**Tll1**	1,33±0,1	2,55±0,6*	2,23±0,1**

Regulation of BMP modulators at 10, 14, and 20 hr after interdigital implantation of a bead bearing TGF-β1. In all experiments an ectopic digit was present in at least 3 out of 4 limbs allowed to develop for 4 days.

* p<0.05;

** p<0.01;

*** p<0.001.

## Results

### Extracellular BMP Modulators

**Figure 1:**
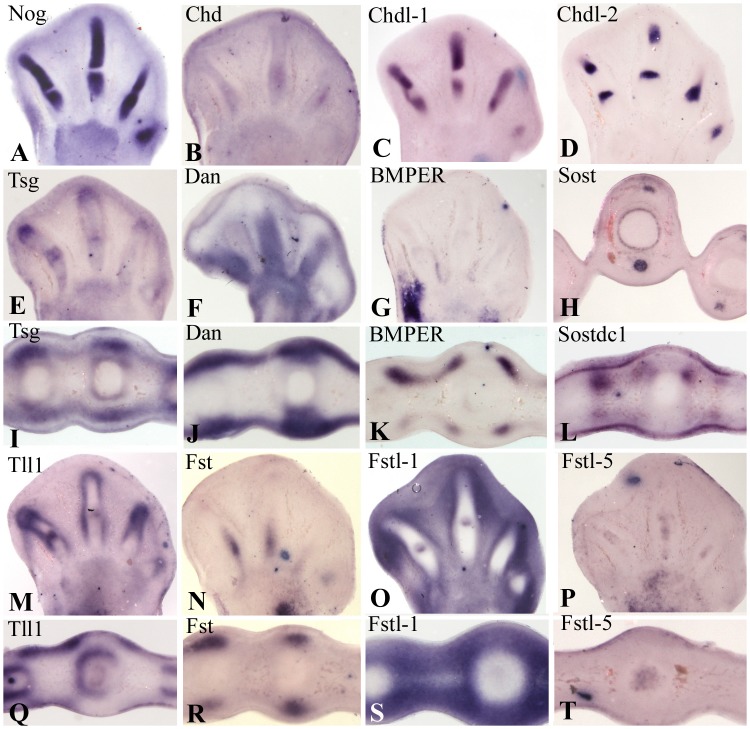
In situ hybridizations in longitudinal (A–G, M–P) and transverse (H–L; Q–T) vibratome sections of the autopod showing the expression of *Noggin* (A), *Chd* (B), *Chdl-1* (C), *Chdl-2* (D), *Tsg* (E and I), *Dan* (F and J), *BMPER* (G and K), *Sost* (H), *Sostdc1* (L), *Tll1* (M and Q), *Fst* (N and R), *Fstl-1* (O and S), and *Fstl-5* (P and T). All the specimens are at day 6,5 of incubation (stage 30HH) except H, which is at 7,5 days of incubation (stage 32HH).

In an initial PCR expression screening, we identified 13 different extracelular modulators of BMP signaling that were expressed at high levels in mesodermal tissues of the developing chick autopod at day 6 of incubation, including: *Noggin*, *Chordin (Chd*), *Chordin-like 1* (*Chdl-1*; *ventroptin*), *Chordin-like 2* (*Chdl-2)*; *Twisted gastrulation* (*Tsg*; *Twsg 1*); *DAN* (*differential screening-selected gene aberrative in neuroblastoma*); BMPER (*BMP binding endothelial regulator; Crossveinless 2*), *Sost* (*Sclerostin*), *Sostdc1* (*Sclerostin domain containing-1; Uterine sensitization associated gene-1; Wise; Ectoidin*), *Follistatin (Fst)*, *Follistatin-like 1 (Fstl-1; Flick*), *Follistatin-like 5 (Fstl-5)*, and *Tolloid* (Tll1;*Colloid*; *Tolloid-like 1*). Although Tolloid is not a BMP antagonist but rather is a protease, it was included here because it is responsible for the release of the BMP ligands that are bounded to BMP antagonists into the target tissues [26]. Although the presence of some of these factors was known from previous studies, we next performed a systematic study of all of them using in situ hybridization to demonstrate a complete picture of their spatial distribution within the developing autopod (Fig. 1). We excluded the members of the CCN secreted factors and the members of the HtrA serine proteases from the study because they have been analyzed in detail elsewhere [44], [47].

**Figure 2:**
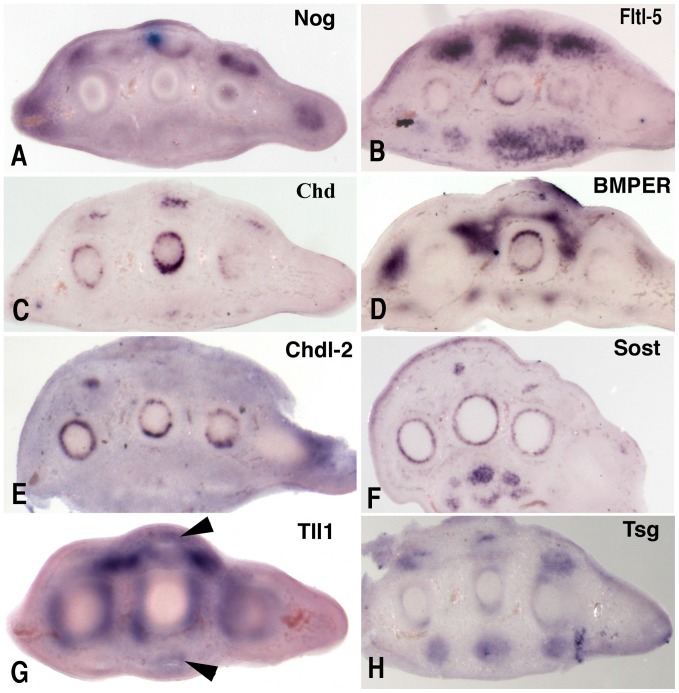
In situ hybridization on transverse sections of the proximal segment of the autopod of embryos after 7 and 7,5 days of incubation, to show the expression domains of BMP modulators in the zone of hypertrophic differentiation of the metatarsal and in the maturing tendons at the zone close to the myotendinous junction. *Noggin* (A), *Fstl- 5* (B), *Chd* (C), *BMPER* (D), *Chdl-2* (E), *Sost* (F), *Tll1* (G), and *Tsg* (H). Note that at this stage of differentiation *Tll1* transcripts are present in the tendon blastemas (arrow heads in G). Note also that the expression domains in the diaphysis of the metatarsal are specifically located in the inner cellular layer of the perichondrium in the interface with the hypertrophic cartilage.

*Noggin* and *Chdl-1*, are prominently expressed in the central region of the differentiating phalanges, excluding the peripheral subperichondrial region and the zones of joint formation (Fig. 1 A and C). *Noggin* is also expressed in the proximal region of the tendons at advanced stages of differentiation (Fig. 2A).

*Chdl-2*, is expressed in the hyaline articular cartilage of mouse embryonic and in human adult osteoarthritic joints [29]. Here we found a wide and well-defined expression domain for this BMP antagonist in the digit blastemas that precede the identification of interphalangeal joints (Fig. 1D). The expression of *Chdl-2* is also noted in the diaphysis of metatarsals preceding the ossification of hypertrophic cartilage forming a collar under the perichondrium (Fig. 2E).

At the beginning of digit formation, *Chd* is expressed at low levels in the digit rays (Fig. 1B), with a slight intensification in the zones of joint formation. Small *Chd* expression domains are also prominent in the zone of the tendons located close to the foot muscles (Fig. 2C). *Chd* transcripts are also present in the diaphysis of the digit cartilages preceding the ossification of hypertrophic cartilage forming a collar under the perichondrium (Fig. 2C).

*Tsg* is expressed in zones of cartilage and tendon differentiation (Fig. 1 E and I). In the chondrogenic regions *Tsg* forms a tenuous expression domain in the cartilage subjacent to the perichondrium of the digit rays. This peripheral digit expression domain is intensified in the digit tip marking the zone of recruitment of cartilage progenitors, the previously termed, “digit crescent” [48], and also in the developing joints. Additionally, its expression is remarkable in the subectodermal mesenchyme with zones of high intensity marking the tendon blastemas.

*Dan* forms a continuous expression domain in the mesenchyme subjacent to the dorsal and ventral ectoderm with zones of increased expression marking the tendon blastemas (Fig. 1 F and J). *BMPER* is expressed in small but intense domains located in the most proximal zone of the interdigits and along the lateral margins of the extensor and flexor tendons (Fig. 1, G and K). It is also strongly expressed in the lateral margins of the autopod at the borderline with the zeugopod. *BMPER* is also expressed in the diaphysis preceding the ossification of hypertrophic cartilage forming a collar under the perichondrium (Fig. 2D).

*Sost* expression has been studied in early stages of limb development [34]. Here we show that at advanced stages of digit development, *Sost* is expressed in the maturing tendons (Fig. 1H) and in the subperichondral region of the diaphysis, which is undergoing hypertrophic differentiation (Fig. 2F).

*Sostdc1* is expressed mainly in the ectoderm with the highest transcript levels in the interdigit region (Fig. 1 L). Transcripts are also observed in the mesenchymal peridigital tissue in a fashion resembling that of *BMPER* (Fig. 1L).

*Tll1* is expressed in the contour of the immature phalanges, including the early developing joints and the tip of the digit (Fig. 1, M and Q). In this distal digit region, the transcripts form a cap encompassing the condensing mesenchyme. *Tll1* transcripts are also present in the mesoderm subjacent to the dorsal and ventral ectoderm of the interdigital and digital regions excluding the zone of tendon formation (Fig. 1Q). However, in the proximal regions where tendon maturation is advanced, including the zone of myotendinous junction, *tll1* transcripts are also detected in the tendon tissue (Fig. 2G).

*Fst* shows restricted expression domains in the tendon blastemas (Fig. 1 N and R). *Fstl-1* is highly expressed in the interdigital mesoderm with additional domains in the developing joints (Fig. 1 O and S), while *Fstl-5*, is expressed at low levels in the core of the differentiating digit cartilages (Fig. 1 P and T). However, in proximal regions where tendon maturations is advanced, *Fstl-5* transcripts are also detected in the tendon tissue and in the subperichondral region of the diaphysis undergoing hypertrophic differentiation (Fig. 2B).

### BMP Signaling Domains in the Developing Digits

**Figure 3:**
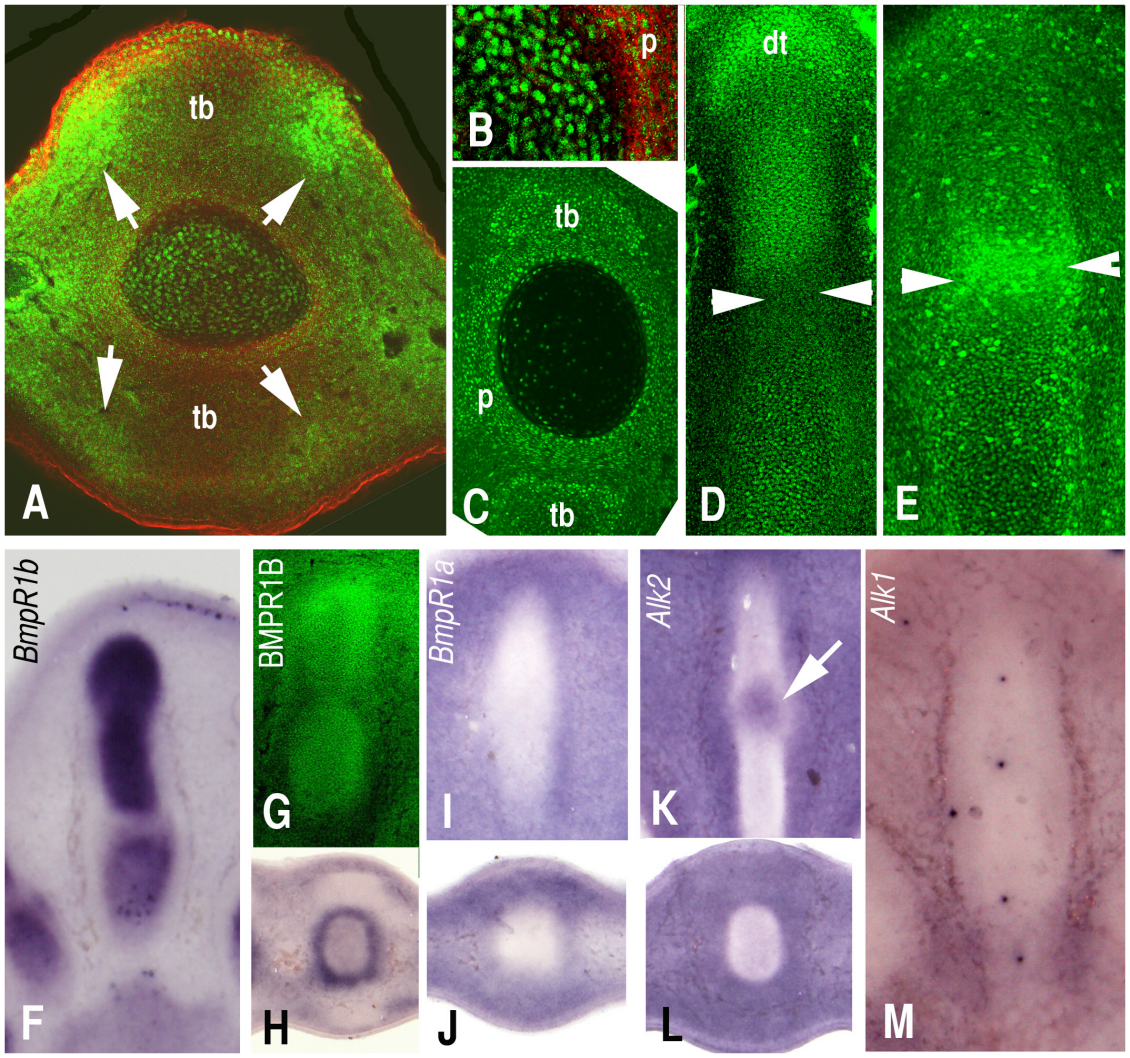
Immunolabeling for p-Smad 1/5/8 (green, A–D) and p-c-Jun (green, E), to show the zones of active BMP signaling in the course of digit formation. Note in A, the absence of labeling in the tendon blastema (tb) at 7,5 days of incubation. In addition, zones of intense labeling paralleling the expression of BMPER of are observed, (arrows, compared with Fig. 1K). B is a higher magnification image of A to show the labeling in the differentiating cartilage except in the most peripheral region subjacent to the perichondrium (p). C shows positive labeling in a proximal region of the tendons corresponding with the myotendinous junction (day 8 of incubation). D is a longitudinal section of digit 3 at day 6 of incubation showing intense labeling in the growing tip (dt) and reduced labeling in the zones of joint formation (arrow heads). E, is a longitudinal section of digit 3, showing intense labeling for p-c-Jun in the joint forming region (arrow heads). In A and B the actin cytoskeleton was labeled in red with Phalloidin-TRITC. F–M, are longitudinal (F, G, I, K, and M) and transverse (H, J, and L) sections through digit 3 showing the expression of type 1 BMP receptors in the autopod. *Bmpr1b* (F–H) is highly expressed at RNA (F–H) and protein (G) levels in the early chondrogenic digit ray with zones of reduced expression in the joint forming region. In the non-chondrogenic domains of the autopod, the expression of *Bmpr1b* is predominant in the subectodermal mesenchyme and interdigital regions. Expression of *Bmpr1a* (I–J) and *Alk2* (K–L) is poor in the chondrogenic rays, but transcripts are abundant in the tissues around the digit cartilage including the perichondrium, the interdigital mesenchyme. Transcripts of *Alk2*, are also appreciated in the developing joints (arrow in K). *Alk 1* was only appreciated in the peridigital blood vessels (M).

The zones of active BMP signaling were monitored by BMP effectors immunolabeling (Fig. 3). Active BMP signaling domains that were marked by phospho-smad 1/5/8 are present in the interdigital mesenchyme and in the core of the developing cartilages, with greater intensity in the tip of the growing digit (Fig. 3 A and D). The perichondrium shows poor labeling and the interface between the cartilage and the perichondrium is relatively devoid of signaling (Fig. 3 B). In the course of digit differentiation, the labeling in the cartilage is reduced while it increases in the outer layer of the perichondrium (Fig. 3C). The tendon formation zones are almost negative, but are encompassed at both sides by zones of intense signal in a pattern closely paralleling the expression of *BMPER*, including stronger expression in the dorsal than in the ventral regions of the autopod (compare Fig. 1K and Fig. 3A). However, a weak positive stain is observed in the tendons at advanced maturation stages and in the zone of myotendinous junction (Fig. 3C). As expected from previous studies [22], the zones of joint formation appear as bands of low phospho-Smad 1/5/8 positivity (Fig. 3D), but exhibit intense labeling by phospho-c-Jun, which is activated by the JNK MAP kinase (Fig. 3E).

As shown in Figure 3 (F–M), all the structures of the autopod exhibited transcripts of at least one of the four type 1 receptors of this signaling pathway, including *Bmpr1b* (*Alk6*), *Bmpr1a* (*Alk3*), *Alk2* (*ACVR1*), and *Alk1* (*ACVRL1*; see [49]), supporting the function of BMP antagonists in the establishment of the zones of active signaling.

### The Expression Sequence of Extracellular Bmp Modulators during the Formation of an Ectopic Digit (Table 1)

To gain insight into the significance of BMP modulators during digit morphogenesis we monitored the temporal sequence of activation of BMP antagonist genes when an ectopic digit is induced in the interdigital regions by implantation of a Tgf β bead [32]. An analysis of expression was performed using Q-PCR at 10, 14 and 20 hr after interdigital implantation of a Tgfβ-bead (Table 1). We selected these time points because 10 hr marks the stage at which genes encoding for the cartilage matrix become upregulated, and 20 hr corresponds with the period when the extra digit condensation become identifiable by specific histological dyes (i.e., Alcian Blue; [44]). At this period the connective tissues located around the ectopic cartilage; including the perichondrium and the pretendinous aggregates, initiates differentiation. *Chdl-1*, *Chdl-2* and *Fst* genes were upregulated at 10 hr after bead implantation, coincident with the upregulation of the most precocious extracellular matrix markers of cartilage differentiation [44]. By14 hr after bead implantation *Noggin*, *BMPER*, and *Tll1* were up-regulated. *Dan* was the last of the examined BMP antagonists to be up-regulated during the formation of the ectopic digit (20 hr after bead implantation), consistent with a function in the differentiation of tendons. In the period covered by our study, *Tsg* appeared moderately upregulated from 10 hr, but without reaching levels of statistical significance. *Fstl-5* was transiently downregulated at 10 hr after bead implantation. There were no changes in the expression of *Fstl-1*, *Sost* and *Sostdc1*. Transcripts of those three genes are present in the interdigital mesoderm from the beginning of the experiment, and the formation of an ectopic digit may not generate detectable changes in their levels of expression.

### Gene Regulation after Administration of Extracellular BMP Modulators

The structure and functional properties of the BMP modulators are not uniform. Antagonism of BMP signaling may be variable due to differential affinity for distinct ligands. Hence, to gain insight about their role in the developing digits, we monitored the effects on the regulation of most of BMP modulators by the addition of recombinant proteins to Micromass cultures of digit progenitors. In some cases, protein-addition based experiments were complemented or substituted by overexpression approaches, employing vectors containing the full gene coding region. Several preliminary experiments were performed to adjust the dose of BMP regulators to obtain clear and reproducible effects on the genes explored. Tables 2 to 4 summarize the changes in the expression of the cartilage (Table 2), joint (Table 3), and tendon (Table 4) markers selected in this study. The effects of BMP2 on the expression of the mentioned markers were also analyzed to allow a clear distinction between effects that were dependent or independent of the inhibition of BMP signaling (data are shown at the bottom of each table). The following aspects can be emphasized from results:

**Table 2:** Regulation of Cartilage Markers.

CARTILAGE	*Sox9*	*Col2α1*	*BmpR1b*
**TSG**	0,99±0,2	1,06±0,1	0,85±0,0
**TSG o/e**	0,38±0,0**	0,46±0,0*	0,58±0,0*
**NOGGIN**	0,27±0,0***	0,11±0,0***	0,16±0,0***
**NOGGIN+TSG**	0,14±0,0**	0,06±0,0***	0,06±0,0***
**CHRDL1**	0,73±0,0	0,96±0,0	1,01±0,0
**CHRDL1+ TSG**	0,37±0,1**	0,44±0,0**	0,23±0,1**
**CHRDL2**	0,77±0,0	0,80±0,0	0,96±0,1
**CHRDL2+ TSG**	0,62±0,0*	0,45±0,0**	0,44±0,0**
**DAN**	1,00±0,1	0,99±0,0	0,96±0,0
**DAN o/e**	0,54±0,0*	0,45±0,0**	0,35±0,0**
**BMPER o/e**	0,56±0,0*	0,45±0,0*	0,80±0,1
**FST**	0,58±0,1***	1,67±0,3*	1,02±0,0
**FSTL1 o/e**	0,35±0,0*	0,39±0,0*	0,73±0,0*
**BMP2**	5,58±0,9*	12,2±2,6*	4,15±0,1*

Regulation of 3 selected markers of cartilage in 2 days Micromass cultures of digit progenitors treated for 24 hr with recombinant proteins. As indicated in the table (o/e), in the cases of TSG, DAN, and BMPER gain-of-function experiments were also performed by transfection of mesodermal cells with expression vectors.

* p<0.05;

** p<0.01;

*** p<0.001.

**Table 3:** Regulation of Joint Markers.

JOINT	*Gdf5*	*Activin BA*	*Jaws*
**TSG**	0,79±0,0*	0,79±0,0	1,22±0,1
**TSG o/e**	0,39±0,0**	0,91±0,1	0,57±0,0*
**NOGGIN**	2,23±0,5*	1,09±0,1	1,38±0,1
**NOGGIN+TSG**	1,82±0,0**	0,80±0,1	0,33±0,0**
**CHRDL1**	1,16±0,1	0,92±0,0	1,01±0,0
**CHRDL1+ TSG**	0,99±0,0	0,44±0,0***	0,85±0,1
**CHRDL2**	1,41±0,2*	0,60±0,0*	0,95±0,1
**CHRDL2+ TSG**	1,45±0,1*	0,83±0,1	1,11±0,1
**DAN**	1,25±0,0	1,11±0,0	0,78±0,0
**DAN o/e**	0,67±0,2	1,18±0,1	0,97±0,2
**BMPER o/e**	1,07±0,1	1,48±0,0***	1,10±0,2
**FST**	1,28±0,1	0,97±0,1	1,31±0,1
**FSTL1 o/e**	1,39±0,0*	0,63±0,0*	1,12±0,1
**BMP2**	0,02±0,0**	12,05±1,2**	1,07±0,2

Regulation of 3 selected markers of fibrous and tendon tissue in 2 days Micromass cultures of digit progenitors treated for 24 hr with recombinant proteins. As indicated in the table (o/e), in the cases of TSG, DAN and BMPER gain-of-function experiments were also performed by transfection of mesodermal cells with expression vectors.

* p<0.05;

** p<0.01;

*** p<0.001.

**Table 4:** Regulation of Tendon Markers.

TENDON	*Scleraxis*	*Col1α1*	*Tgfβ2*
**TSG**	1,15±0,2	1,12±0,0	0,73±0,0**
**TSG o/e**	0,83±0,0	0,83±0,1	0,52±0,0***
**NOGGIN**	1,98±0,3*	1,50±0,1**	2,97±0,4**
**NOGGIN+TSG**	2,15±0,0**	1,72±0,1**	4,79±0,3**
**CHRDL1**	0,99±0,0	0,98±0,0	0,98±0,1
**CHRDL1+ TSG**	0,60±0,1	0,90±0,1	0,66±0,0*
**CHRDL2**	1,53±0,0*	1,13±0,1	1,59±0,1*
**CHRDL2+ TSG**	1,81±0,3*	1,20±0,2	1,21±0,2
**DAN**	1,28±0,3	1,01±0,0	1,02±0,0
**DAN o/e**	2,64±0,6*	0,94±0,0	0,83±0,0
**BMPER o/e**	2,38±0,2*	1,08±0,0	1,36±0,2
**FST**	0,97±0,1	1,05±0,1	0,93±0,1
**FSTL1 o/e**	1,81±0,1**	1,40±0,0**	1,44±0,0**
**BMP2**	0,13±0,0**	0,69±0,0	0,79±0,0

Regulation of 3 selected genes responsible for joint formation in 2 days Micromass cultures of digit progenitors treated for 24 hr with recombinant proteins. As indicated in the table (o/e), in the cases of TSG, DAN and BMPER gain-of-function experiments were also performed by transfection of mesodermal cells with expression vectors.

* p<0.05;

** p<0.01;

*** p<0.001.

1. The overexpression of the mouse *Tsg* gene induced down-regulation of chondrogenic markers (*Sox9*, *Collagen2 α1*, *Bmpr1b*), *Gdf5* and *Jaws*. In addition, the tenogenic master gene *Tgfβ2*, which was not regulated by BMP2, was strongly downregulated following *Tsg* overexpression.
2. At the highest doses tested (up to 1000 ng/ml) rhTSG, was much less effective than gene overexpression experiments. Treatments caused only a mild downregulation of *Tgfβ2* and *Gdf5*. However, the gene regulation that was induced by CHDL-1, CHDL-2, or NOGGIN was strongly potentiated when they were administered in combination with rhTSG (1000 ng/ml). This effect supports the functional association between TSG and CHD that has been observed in other systems [50] and supports also the interaction of TSG with NOGGIN, which to our knowledge, has not been reported in previous studies. This interplay potentiated the BMP antagonistic effect of CHDL-1 and NOGGIN, and also caused modifications in the regulation of genes not induced by BMP2 (see below), and even regulations not observed in separate treatments of TSG and CHDL-1, or NOGGIN (i.e. the regulation of *Activin βa* in combined treatments with CHDL1-TSG, Table 3; and see below for interactions NOGGIN-TSG).
3. NOGGIN, was the most intense antagonist of gene regulation induced by BMPs in Micromass cultures (see tables 2–4); however, treatments also induced the upregulation of *Scleraxis*, *type 1 Collagen*, and *Tgfβ2* which are not regulated by BMP2. As mentioned above, the effects of NOGGIN were intensified when administered in combination with TSG. Intensification was appreciated even for the effects like the up-regulation of *Scleraxis*, *type 1 Collagen*, or *Tgfβ2* which are not induced by BMP2. In addition, NOGGIN in combination with TSG down-regulate the expression of *Jaws*. A detailed analysis of the molecular bases for the interplay between TSG and NOGGIN is out of the scope of this study. However, considering the roles of TSG in other systems (see discussion for references), a tentative explanation is that the addition of TSG may protect sequestering and/or degradation of NOGGIN in the extracellular matrix.
4. CHDL-1, was ineffective at doses ranging between 400 and 2500 ng/ml; however, as previously mentioned, CHDL-1 in combination with rhTSG exhibited BMP antagonism at remarkable levels.
5. CHDL-2 administered alone (up to 1200 ng/ml), caused a moderate upregulation of *Scleraxis*, *Tgfβ2* and *Gdf5* and had no effect on most chondrogenic markers; however, when CHDL-2 was administered in combination with rhTSG, *Sox9*, *type 2 Collagen* and *Bmpr1b* were downregulated.
6. Previous analysis in different systems has provided controversial information concerning the targets of DAN [38], [51], [52]. It has been proposed that the production of bioactive DAN protein is cell-specific [52]. In our analysis the BMP targets analyzed here were not significantly regulated by mouse recombinant DAN even at doses up to 3000 ng/ml. However, overexpression of the mouse *Dan* gene down-regulated chondrogenic markers (*Sox9*, *type 2 Collagen*, and *Bmpr1b*) and up-regulated the expression of *Scleraxis* more than twofold.
7. BMPER gain-of-function experiments were done by overexpressing the mouse *BMPER* gene and the effects on the chondrogenic markers corresponded largely with those of a typical BMP antagonist. However, *Scleraxis*, and *Activin βa*, which are not regulated in treatments with BMP2, were also up-regulated by this factor.
8. FST exhibited an intense inhibitory effect on *Sox9* gene expression, but the expression of other genes up-regulated by BMPs was not down-regulated. Remarkably, no effects of FST were recognized in tendon markers in spite of its restricted expression in the tendon blastemas.
9. The overexpression of *Fstl-1* had similar, but less intense, effects than the addition of rh-NOGGIN, except for a negative influence in the expression of *Activin βa* which did not occur with NOGGIN.

### Cell Proliferation and Cell Death in Micromass Cultures following Treatments with BMP Antagonists

The low anti-BMP influence on gene regulation in rh-CHDL-1 alone treatments prompted us to check whether it has an effect promoting cell proliferation as reported in cultures of human mesenchymal stem cells [53]. However, no changes in cell proliferation or apoptosis were observed in cultures treated with different doses of CHDL-1 (Fig. supplementary 1).

In view of the functional potentiation of the effects of CHDL-1 when administered in combination with TSG, we further analyzed the effects of the combined treatments of CHDL-1 and TSG. Neither cell proliferation or cell death was modified by this treatment (Fig. supplementary 1).

### Transcriptional Regulation of BMP Modulators by Major Signaling Pathways Operating in the Developing Autopod

To the light of the regulated expression patterns of BMP antagonists and their distinct effects on mesodermal tissue differentiation, we sought to known if, as reported in other models [54], there is an interactive signaling network which establishes the level of expression of the different regulatory components of the pathway, or if additional signaling pathways regulate their expression.

The regulation of BMP modulators was studied in 2 days old Micromass cultures after 6 hr treatments with the signaling molecules that are responsible for growth and differentiation of the autopod mesoderm, including the following: FGFs which are implicated initially in the maintenance of the undifferentiated state of the mesoderm in the distal margin of the bud and in inhibiting the chondrogenic differentiation of digit progenitors [14], and they are involved later in tendon differentiation [55]; all-trans-Retinoic Acid which, similar to FGF signaling, is a primary inhibitor of mesodermal differentiation [56] and later regulates the differentiation of tendons [57]; ACTIVIN A, which is an early signal for the formation of digit chondrogenic aggregates and next a prominent joint marker [40]; TGF βs, which are responsible for the formation of tendons but also promote the formation of prechondrogenic aggregates [58], [59]; BMP2, which together with other BMPs are responsible for cartilage differentiation [8]; and WNT 5a, which together with other members of the family are involved in cartilage and joint differentiation [60].

The following data can be stressed from our results (Table 5):

**Table 5:** Regulation of BMP modulators.

	FGF2	RA	Activin A	TGFβ1	BMP2	Wnt5a
**Noggin**	0,40±0,0*	0,51±0,0***	2,15±0,3*	1,76±0,2*	33,86±2,5**	1,14±0,1
**Chordin**	0,72±0,0	0,12±0,0***	1,35±0,2	0,59±0,0*	0,17±0,0***	0,96±0,1
**Chdl-1**	0,12±0,0**	0,66±0,0**	2,29±0,3*	0,52±0,0*	3,75±0,1***	1,21±0,2
**Chdl-2**	0,05±0,0***	0,01±0,0**	1,90±0,1**	0,67±0,0	0,65±0,2	0,98±0,0
**Tsg**	1,35±0,1	1,04±0,0	2,09±0,1*	0,91±0,0	0,53±0,0***	1,24±0,1
**Dan**	0,15±0,0**	0,54±0,0**	0,46±0,0*	0,83±0,0	0,14±0,0***	1,09±0,3
**Sost**	2,64±0,4*	0,80±0,0	0,63±0,2	0,92±0,1	0,38±0,0*	1,98±0,1*
**Sostdc1**	0,88±0,0	2,66±0,3**	1,18±0,2	3,40±0,2*	0,50±0,0***	1,02±0,2
**Follistatin**	0,98±0,0	0,81±0,0	9,06±0,7**	4,95±0,6*	7,48±0,8**	1,04±0,1
**Fstl-1**	0,33±0,0***	0,90±0,0	1,56±0,0*	1,18±0,3	0,45±0,0*	0,80±0,0
**Fstl-5**	2,37±0,2*	0,83±0,0	0,75±0,1	0,94±0,2	0,66±0,1	1,09±0,2
**Tll1**	0,21±0,0**	0,26±0,0**	0,73±0,0	0,47±0,0*	0,21±0,0***	0,86±0,0
**Bmper**	8,29±0,3**	0,90±0,1	0,32±0,0*	0,40±0,0*	0,34±0,0**	4,57±0,1***

Regulation of BMP modulators in 48 hr Micromass cultures of digit progenitors treated for 6 hr with BMP2, FGF2, all-trans-retinoic acid, TGFβ1, ACTIVIN A, and WNT 5A.

* p<0.05;

** p<0.01;

*** p<0.001.

1. In the FGF treated cultures, the expression of BMP modulators including *Noggin*, *Chordins* and *Fstl-1* was inhibited. The inhibition was also strong for *Dan* and *Tll1*. In contrast, *BMPER*, *Fstl-5*, and *Sost* were highly upregulated by FGF treatments. Other antagonists (*Sostdc1*, *Fst*, and *Tsg*) were not regulated at significant levels by FGFs.
2. Consistent with the functional similarity between retinoic acid and FGF signaling in cartilage and tendon differentiation at the stages studied here, the addition of all-trans- Retinoic Acid (RA) to the culture medium was followed by a downregulation of *Noggin*, *Chordins*, *Tll1* and *Dan*. In contrast with findings obtained by FGF treatments, *Sostdc1* was the only antagonist upregulated by RA. The expression of the remaining studied antagonists was not modified by RA treatments.
3. Treatments with ACTIVIN A resulted in a strong upregulation of *Fst* expressio*n*. There was also an increase in the expression of the BMP modulators *Noggin*, *Chordins*, *Tsg* and *Fstl-1*. In contrast the BMP modulators that are expressed in or around the tendon blastemas, including *Dan*, and *BMPER* were downregulated.
4. Tgfβ1 treatments upregulated *Noggin*, *Sostdc1* and *Fst* while *Chordins*, *Tll1* and *BMPER* were downregulated. The expression levels of the other studied antagonists were not modified.
5. BMP2 treatments induced a strong up-regulation of *Noggin*, *Chdl-1*, and *Fst* while all the remaining antagonists were downregulated. It is remarkable from these findings that BMP2 was the only treatment which downregulated the expression of *Tsg*
6. WNT5a treatments induced up-regulation of *BMPER* and *Sost*. Expression of other BMP modulators was not modified.

## Discussion

We show that 9 representatives of the three recognized subfamilies of BMP antagonists [61] together with *Tll1*, *Fst*, *Fstl-1* and *Fstl-5* are expressed in a regulated fashion during the early histogenesis of the digit tissues. These factors are precociously induced during the formation of an extra digit, preceding the appearance of changes identifiable by conventional histological procedures, such as the establishment of phalanges, joints and tendons. These findings support the role of BMP modulators in digit morphogenesis. However, except for Noggin [4], Fstl-1 [42], and Sost [62], mice with genetic alterations in these factors, including *BMPER* [63], *Tsg* [63], [64], *Chd* [65], *Dan* [38], *Fst* [66], Sostdc1 [67], and *Tll1* [68] lack a digit phenotype. The lack of a phenotype in these mutants is likely explained by functional redundancy [42], [69]. In line with this interpretation, we show overlapping expression of *Noggin/Chdl-1* in the developing cartilage, *Chdl-2/Fstl-1* in the developing joints, *Tsg/Dan/Fst* in the tendon blastemas, *BMPER/Sostdc1/Fstl-1* in the peritendinous mesenchyme, and *Chd/Chdl-2/Fstl-5/BMPER/Sost* under the perichondrium of the diaphysis that is undergoing hypertrophic differentiation and subsequent ossification.

The functional properties of the different BMP antagonists often includes crosstalk or the inhibition of other signaling pathways which results in tissue- and developmental-context dependent responses to their local administration [70], [71]. Consistent with the occurrence of functional specializations, the analyzed antagonists downregulated the expression of BMP target genes during skeletogenesis at different levels and their expression was differentially controlled by the distinct signaling pathways acting in the developing limb at the stages covered by this study (see Table 5). Together these findings favor the view considering that morpho-histogenesis of cartilage/bone, joints, and tendons in the embryonic limb is generated by a cascade of autoactivation and lateral inhibition signals resulting from local interactions of mesenchymal progenitors (“reactor–diffusion” model, see [72], [73], and references therein). The formation of the prechondrogenic aggregate constitutes the first step of this process and is followed not only by the inhibition of chondrogenesis in the adjacent tissue, but also by signals which regulate its divergent differentiation to form joints and tendons.

Considering the pattern of expression in the autopod (Table 6), the different BMP modulators can be grouped as follows: 1) cartilage associated, which include: *Noggin*, *Chdl-1*, and *Fstl-5*; 2) joint associated, represented by *Chdl-2* and *Fstl-1*, although the later is also expressed in the undifferentiated interdigital mesenchyme, and *Noggin* is expressed in the cartilage encompassing the developing joints; 3) tendon or associated peridigital connective tissue, which include the following: *Dan*, *BMPER*, *Sos*t, *Sostdc1*, *Fst*, and at stages of advanced differentiation, also *Noggin, and Fstl-5*; and, 4) a group which can be termed “mixed distributed”, is represented by *Tsg*, *Tll1*, and *Chd* which may function in concert with the other antagonists.

**Table 6:** Semi-quantitative association of gene expression intensity in the autopodial tissues.

	Cartilage	Joint	Early Tendon	Late Tendon	Peri-tendinous
**Noggin**	+++	–	–	+	–
**Chordin**	+	++	–	+	–
**Chdl-1**	+++	–	–	–	–
**Chdl-2**	–	+++	–	–	–
**Tsg**	+	++	+++	+	+
**Dan**	–	–	+++	++	++
**Bmper**	–	–	–	–	+++
**Sost**	–	–	–	++	–
**Sostdc1**	–	–	–	–	++
**Follistatin**	–	–	+++	++	–
**Fstl-1**	–	++	+	+	++
**Fstl-5**	+	–	–	+++	–
**Tll1**	+	++	–	+	++

Table summarizing the expression intensity of the BMP modulators in the autopodial tissues.

### TSG, Chd, and Tll1 and the Differentiation of Cartilage and Tendons

Due to their association with other BMP modulators in developing cartilage, joints and tendons, the functional significance of TSG, CHD, and TLL1, merits individual discussion. TSG is a multifunctional BMP modulator, which interacts with other antagonists to potentiate or inhibit their function depending on the proteolytic activity of TLL1 (reviewed in [74]). TSG forms heterotrimeric complexes with BMPs and CHD which potentiates the antagonistic effect of CHD and facilitates the diffusion of BMP ligands through the extracellular space to reach the appropriate targets [28], [75]. In addition, when the complex is subjected to the action of TOLLOID metalloprotease, CHD is cleaved, delivering active BMPs [76]. TSG also forms trimolecular complexes with BMPER and BMP4 [69] but in this case the complex does not promote BMP diffusion [26].

We show that the contour of the phalanges at the stages of initial differentiation express *Tsg*, *Tll1* and *Chd*. Taking into account that BMP ligands are predominantly expressed outside the cartilage rods in the undifferentiated interdigital mesoderm [3], this expression pattern is consistent with a function of the complex TSG/CHD in the transport and subsequent delivery of BMPs into the differentiating cartilages, which are positive for phospho Smad 1/5/8 immunolabeling. In our culture model, the overexpression of *Tsg* down-regulates markers that are activated by BMPs. In addition, consistent with previous studies [64], [77], [78], we also noted the occurrence of pro-BMP responses, such as the intense down-regulation of *Gdf5*, which is an effect that is also induced by BMP2 treatments.

The distribution of “mixed distributed” BMP modulators in the zones of tendon formation is more complex. We show that the core of the tendon blastemas expresses high levels of *Tsg* accompanied, in a stage-dependent manner, with other BMP antagonists (see discussion below). Remarkably, at initial stages of formation, the tendon blastemas lack *Tll1* transcripts, suggesting that in this tissue TSG exerts only an anti-BMP function. This interpretation is consistent with the poor labeling of the tendon blastemas with phospho Smad 1/5/8. Taking into account that overexpression of TSG in mesoderm progenitor cultures results in the inhibition of chondrogenic markers without a positive influence on tendon markers, the expression of *Tsg* could preclude chondrogenic differentiation of the pretendinous mesenchymal aggregates, which is the default fate of the undifferentiated limb mesoderm. Mice deficient in TSG lack a digit or tendon phenotype [64], but this could be explained by the overlapping expression of additional antagonists (see below).

During the differentiation of the tendon blastemas, in the subectodermal mesenchyme of the proximal region of the interdigit intercalated between two neighbor tendons, there are intense expression domains of *Tll1* overlapping with low levels of *Tsg* and high levels of *BMPER*. This gene expression pattern correlates with strong immunolabeling for phospho Smad 1/5/8, suggesting that the presence of TOLLOID reverses the influence of TGS on BMP signaling from negative to positive. Furthermore, the negative regulation of *Tll1* by FGFs, TGF βs, and RA which are all key signals in the differentiation of tendons [55], [57]–[59] reinforces the idea that TSG functions in the initial differentiation of the tendon blastemas requires the absence of TOLLOID. At more advanced stages of differentiation and coinciding with the appearance of *Tll1* transcripts, immunolabeling for phospho-Smad 1/5/8 becomes visible in the tendon primordia.

### BMP Antagonists and the Formation of Phalanges and Joints

Digits develop as cartilage rods, which become segmented into jointed structures by local de-differentiation of cartilage. Hence, the zones of joint formation appear as strips of fibrous-like connective tissue, which constitute the substrate for subsequent cavitation and differentiation of the joint tissues (fibrous capsule and synovium). It has been shown that Jaws exerts a central role in the formation of joints, although lack specific expression domains in these regions [45]. The formation of phalanges is a direct effect of BMP signaling via Smad 1/5/8 [48]. Differentiation of the joints is also controlled by BMP signaling [5], [79], but it is directed by the activation of MAP kinases [22]. Furthermore, the reduced phalangeal size and loss of interphalangeal joints that is observed in humans and mice deficient in NOGGIN [4], [17], indicates that regulation of BMP signaling is a central function in the formation of phalanges and joints.

Our findings reveal that the outer layer of the developing phalanges expressing *Tsg*/*Tll1*/*Chd* encompass a core of differentiating chondrocytes expressing *Chdl-1* and *Noggin* in the body of the phalanx, and *Chdl-2* in the zones of joint formation. We show that CHDL1 alone, or even in combination with TSG, does not modify proliferation, at difference of studies in other systems [53]. The effect of NOGGIN and both CHDL-1 and CHDL-2 on cultures of digit progenitors, concerns tissue differentiation, and becomes intensely potentiated by TSG. Together these findings suggest that, in addition to the previously discussed role of TSG/CHD complexes in the transport of BMP ligands, TSG also functions in a concerted fashion with NOGGIN and CHORDINS to modulate the intensity of BMP signaling in the differentiating cartilage.

We further show that cartilage-expressed *(Noggin* and *Chdl-1*) and joint-expressed antagonists (*Chdl-2* and *Fstl-1*) are regulated in an opposite fashion by BMP2. The positive regulation of *Noggin* and *Chdl-1* by BMP2 is consistent with the occurrence of a negative feed-back loop tuning the level of BMP signaling in the differentiating cartilage as observed in different systems [80]. Conversely, the negative regulation of *Chdl-2* by BMP2, suggest that joints are formed in zones of the digit cartilage templates with the lowest BMP signal.

### BMP Antagonists and the Establishment of Tendons and Intertendinous Mesoderm

Tendon blastemas are formed in the mesoderm subjacent to the dorsal or ventral ectoderm. We have previous shown that all the subectodermal tissue of the autopod has the potential to differentiate into tendons, but that in normal conditions, tendons differentiated only in the digit regions [40], [81]. The present study shows a regionalization of the mesoderm subjacent to the ectoderm into digit and interdigit regions characterized by distinct distribution of BMP antagonists accompanied, at the beginning of tendon formation, by differences in Smad 1/5/8 phosphorylation. Tendon blastemas are formed in zones expressing high levels of *Dan*, *Fst*, and *Tsg*, which recruit *Chd*, *Noggin* and *Sost* at advanced stages of differentiation. During the differentiation of these tendon-forming regions, the subectodermal mesenchyme intercalated between the tendon blastemas shows intense domains of *BMPER* expression accompanied by reduced expression of *Dan*, *TSG*, *Tll1*, and *Sostdc1*. As mentioned above, the different patterns of gene expressions correlate with a dramatic change in the intensity of BMP signaling. This finding might be of interest in regenerative medicine to direct the differentiation of connective tissue progenitors. However, there is no tendon phenotype in mice deficient for these factors [38], [63], [66], [67]. The difference in the intensity of phospho Smad 1/5/8 immunolabeling might be due to the antichondrogenic and profibrogenic action of DAN in conjunction with and the functional interplay between BMPER with TSG and TLL1. BMPER has been characterized as a CHD related BMP modulator with context-dependent pro-BMP or anti-BMP activities [63], [69], [82]–[84]. Our findings show that its expression marks zones of very high BMP activity. In previous studies the expression of BMPER in the autopod has been functionally related with interdigital cell death [33]. However, the spatial distribution of BMPER transcripts and their maintenance after the period of interdigital cell death, support the involvement of this BMP modulator in the formation of the peridigital connective tissues.

### Conclusion

In conclusion our findings reinforce the morphogenetic importance of BMP antagonists in the establishment of a molecular signaling scaffold that is responsible for the allocation of the cell fate of digit mesodermal progenitors. The information drawn from this study provides a basic view of this functional signaling network but further work is required to unravel the exquisite extracellular regulation of BMP signaling during the histotypic differentiation of digit precursor mesoderm. A subject to be addressed in future studies is that the function of the different BMP modulators in tendon development may result from a combination BMP and Wnt modulation, as several factors such as Sost [34], [85], Noggin [86], Chd [87], and Sostdc1 [85], [88], have been shown to exert both functions.

## Supporting Information

Figure S1 **Representative flow cytometry plots of dissociated mesodermal cells propidium iodide stained, obtained from 2 day Micromass in control (A and B), CHDL-1 treated (A′ and B′), and CHDL-1 plus TSG treated cultures of digit progenitors.** Upper panels (A–A″) represent the cell cycle distribution of cells expressed in a linear scale. In lower panels (B–B″) the intensity of propidium iodide label is plotted on a logarithmic scale to show the presence of cell death (sub-G1 region). The percentage of dying cells is indicated.
(TIF)

Table S1 **Primers for Q-PCR.** Note that except indicated, the primers are for *Gallus gallus*.
(DOC)
