# Supplementary figures and images for: Expression and Functional Study of Extracellular BMP Antagonists during the Morphogenesis of the Digits and Their Associated Connective Tissues
Source: PLoS One. 2013 Apr 3;8(4):e60423. doi: 10.1371/journal.pone.0060423 (PMC3616094; doi:10.1371/journal.pone.0060423)

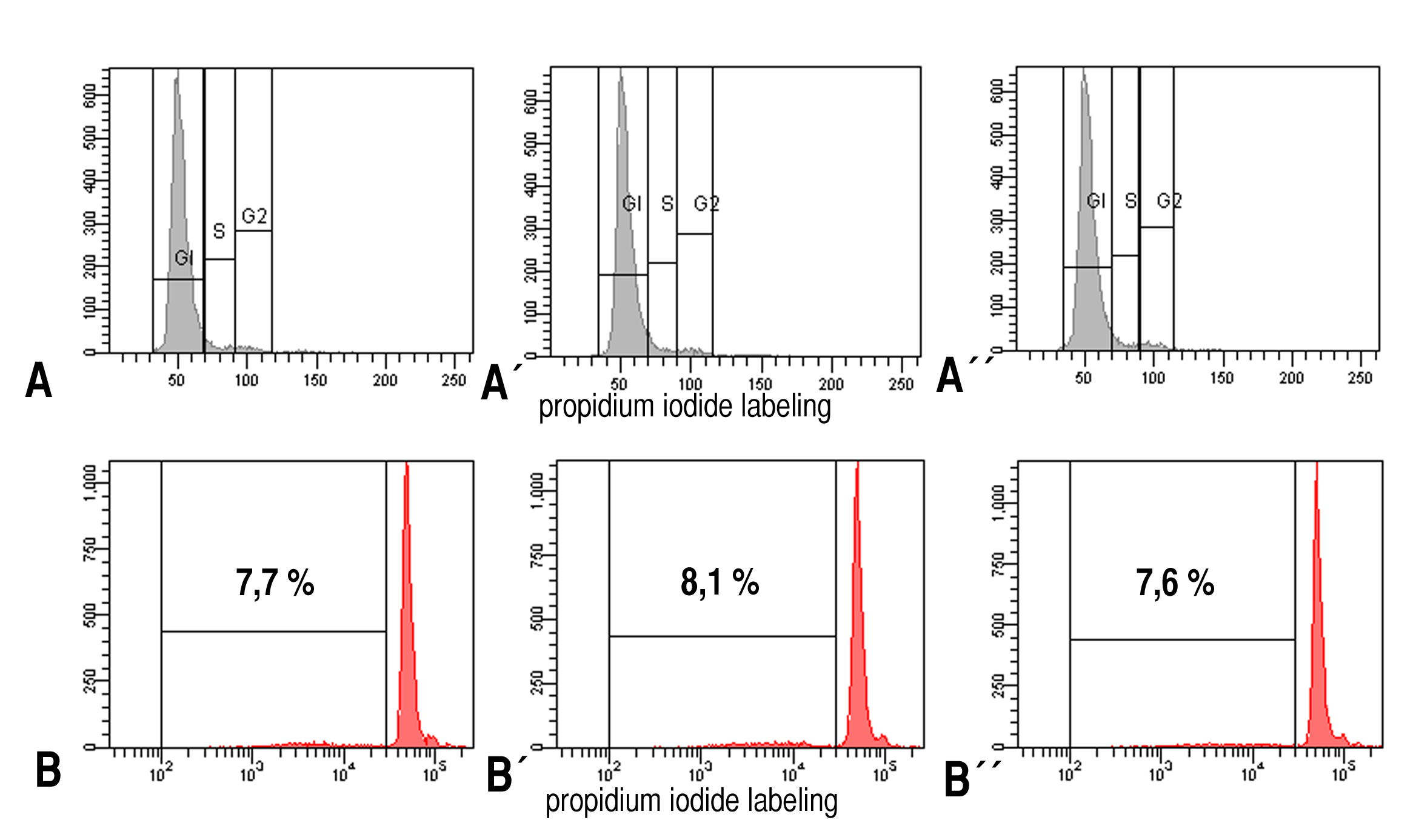

Supplement: Figure S1 — Representative flow cytometry plots of dissociated mesodermal cells propidium iodide stained, obtained from 2 day Micromass in control (A and B), CHDL-1 treated (A′ and B′), and CHDL-1 plus TSG treated cultures of digit progenitors. Upper panels (A–A″) represent the cell cycle distribution of cells expressed in a linear scale. In lower panels (B–B″) the intensity of propidium iodide label is plotted on a logarithmic scale to show the presence of cell death (sub-G1 region). The percentage of dying cells is indicated. (TIF) [file pone.0060423.s001.tif]
