# Supplementary material for: Expression and Functional Study of Extracellular BMP Antagonists during the Morphogenesis of the Digits and Their Associated Connective Tissues
Source: PLoS One. 2013 Apr 3;8(4):e60423. doi: 10.1371/journal.pone.0060423 (PMC3616094; doi:10.1371/journal.pone.0060423)
Supplement: Table S1 — Primers for Q-PCR. Note that except indicated, the primers are for Gallus gallus. (DOC) [file pone.0060423.s002.doc]

| **Gene** | **GenBank** | **Forward Primer 5’- 3’** | **Reverse Primer 5’- 3’** |
| --- | --- | --- | --- |
| ***Gapdh*** | NM_204305 | ggtggccatcaatgatcc | gttctcagccttgacagtgc |
| ***Noggin*** | NM_204123 | ccagcactacctgcacatcc | ggctccttagcaaggtctcg |
| ***Chd*** | AF031230 | cgctggtacctcaacaacg | tgacacacaccagcacagg |
| ***Chdl-1*** | NM_204171 | tccaagtgccaggagtaacc | aactcgtccatgcttgtgc |
| ***Chdl-2*** | XM_417245 | caagaagcgcagaactacagg | tgtcatcctcacctctgacg |
| ***Tsg*** | NM_204198 | cgttgcagaagagctgtcg | gagacgttctgatgctgtgg |
| ***Dan*** | NM_204149 | aatgtctacttgaacgcagagg | ggctcttctacctcctgttgg |
| ***BMPER*** | NM_001007080 | gctgcaaggaatgtgtctcc | gatggtgcagttcacagagg |
| ***Sost*** | XM_427339 | agtggtggaggcagaactcc | taccgagtgtagcgcttgc |
| ***Sostdc1*** | NM_204373 | agcaacagcacgttgaacc | acttgaacgcgattgttacg |
| ***Fst*** | NM_205200 | acttatccgagcgagtgtgc | gagttgcaagatccagagtgc |
| ***Fstl-1*** | NM_204638 | gaatgtgcagtgactgagaagg | ccattgctaccacacacagg |
| ***Fstl-5*** | XM_426284 | aggcactgtgtcatgaatgg | catctgagccacacacagg |
| ***Tll1*** | NM_204703 | tatgtggagattcgcagtgg | aacttcaggcacttccgtacc |
| ***Sox9*** | NM_204281 | gaggaagtcggtgaagaacg | gatgctggaggatgactgc |
| ***Col2α1*** | NM_204426 | cagcatccagatgaccttcc | gtctcctcgtccatgtaggc |
| ***BmpR1B (ALK6)*** | NM_205132 | cctcctacgaggacatgagg | gcactccatcatgagcttcc |
| ***Scleraxis*** | NM_204253 | caccaacagcgtcaacacc | cgtctcgatcttggacagc |
| ***Col1α1*** | M10571 | ttgtggattctcggttactgc | ggtctggatgtcctcttctcc |
| ***Tgf*β*2*** | XM_003640970 | tgcactgctatctcctgagc | gcatgaactgatccatgtcg |
| ***Gdf5*** | NM_204338 | acctgaagccaaggtgtagc | agaccttcgcagtgatacgc |
| ***Activin* β*A*** | NM_205396 | gctgactgtccatcatgtgc | actgcttccaccatctcagg |
| ***Jaws*** | XM_419214 | tctgaggaacgtgttgatgc | tcttcaggaatactgcgatcc |
| ***Human Fstl-1*** | NM_007085 | cacctgtctctgcattgagc | gcagttcacagtggttgagg |
| ***Mouse Tsg*** | NM_023053 | ctcggaattacagcgacacc | gagacgatgttccagttcagc |
| ***Mouse BMPER*** | NM_028472 | atccaacgaattctgcaacc | tcactgtgccttgacagagc |
| ***Mouse Dan*** | NM_008675 | ccgccacctatcaacaagc | ggatagacttggcctcacagc |
